# Safe reopening of university campuses is possible with COVID-19 vaccination

**DOI:** 10.1371/journal.pone.0270106

**Published:** 2022-07-21

**Authors:** Matthew Junge, Sheng Li, Samitha Samaranayake, Matthew Zalesak

**Affiliations:** 1 Department of Mathematics, Baruch College, New York, New York, United States of America; 2 School of Public Health, City University of New York, New York, New York, United States of America; 3 School of Civil and Environmental Engineering, Cornell University, Ithaca, New York, United States of America; 4 School of Operations Research and Information Engineering, Cornell University, Ithaca, New York, United States of America; Centers for Disease Control and Prevention, UNITED STATES

## Abstract

We construct an agent-based SEIR model to simulate COVID-19 spread at a 16000-student mostly non-residential urban university during the Fall 2021 Semester. We find that mRNA vaccine coverage at 100% combined with weekly screening testing of 25% of the campus population make it possible to safely reopen to in-person instruction. Our simulations exhibit a right-skew for total infections over the semester that becomes more pronounced with less vaccine coverage, less vaccine effectiveness and no additional preventative measures. This suggests that high levels of infection are not exceedingly rare with campus social connections the main transmission route. Finally, we find that if vaccine coverage is 100% and vaccine effectiveness is above 80%, then a safe reopening is possible even without facemask use. This models possible future scenarios with high coverage of additional “booster” doses of COVID-19 vaccines.

## 1 Introduction

During March 2020 of the COVID-19 pandemic most universities in the United States halted on-campus operations and went to remote instruction. About one third reopened to full, or partial in-person instruction in Fall 2020, with more schools following suit in the Spring [1, 2]. Many reopenings were accompanied by infection spikes which required temporary pivots to remote instruction [3, 4]. Near the end of the school year (April 30 2021), the New York Times reported over 660,000 confirmed cases of COVID-19 on college campuses. These are directly linked to over 100 deaths, mostly involving employees [5].

Human daily behavioral contrats are primary among similar age groups and secondarily with other age group family members. A big challenge introduced by reopening schools is the restoration of same age social contacts. A previous study has shown school closure and social distancing dramatically reduce daily physical contacts, particularly the dominant same age group contacts. This efficiently prevents the COVID-19 transmission [6].

Universities that reopened to in-person instruction in Fall 2020 implemented protocols to help control infection spread such as: periodic testing, mandatory facemask use, social distancing, building closures, limited extracurricular activities, and hybridized in-person/remote classroom instruction. As these levels of intervention lacked much precedent, various models were developed to help guide policy and predict outcomes [7, 8]. Policy decisions ultimately struck a balance between forecasts, campus safety and comfort, and university resources [9].

With the introduction of apparently effective vaccines [10–13] and increased natural immunity from earlier exposure [14], more universities are conducting Fall 2021 primarily in-person [15]. However, heightened prevalence of the delta (B.1.1.7) variant has resulted in increased uncertainty surrounding how much intervention is needed to control COVID-19 infections. Accordingly, we develop an agent-based SEIR model to forecast total COVID-19 infections over the course of a semester at a primarily non-residential urban university campus with 16000 students and 800 faculty. Baruch College, part of the over 275,000 student City University of New York (CUNY), is used to inform our framework.

Urban universities, such as those in the CUNY system, are usually located in densely populated areas and serve many students from minority groups. Surveys indicate that vaccine hesitancy from minority groups, that present higher COVID-19 infection incidence and higher than average vaccine hesitancy, will limit vaccine coverage to somewhere between 60-80% of the United States population with African Americans among the most hesitant [16–20]. As many students live with their families, reopening such universities is accompanied by elevated risk to and from their households and communities.

## 2 Methods

We model different scenarios with two primary variables: *vaccine effectiveness* and *vaccine coverage* of the campus population. We utilize the agent-based campus Susceptible-Exposed-Infected-Removed model from [21]. Similar to [7, 8], students and faculty are assigned individualized schedules that they follow throughout a simulated semester. Schedules are organized into common meetings—classroom, broad environment, clubs, residential, socializing—during which COVID-19 is equally likely to be passed from infected agents present to each susceptible agent also present. The infection rate in our model is set to obtain an *average reproduction number* *R*_0_ = 3, which represents the average number of infections caused by an exposed agent in an entirely susceptible population. Estimates for COVID-19 spread in large communities (such as cities and countries) put *R*_0_ for the alpha variant in the range [1.5, 3.0] [22–26]. It is believe that the delta variant is more infectious [27]. Additionally, the statistic varies by community contact structure. It has been observed that *R*_0_ is larger in reopened universities [4]. For example, [7] sets *R*_0_ = 3.8 in their campus COVID-19 model for a mostly residential urban university. See [7, 8] for more discussion about elevated *R*_0_ levels in a university setting. We remark that our model implicitly assumes agents are wearing facemasks while on campus. The assumption is implicit because we base our choice of *R*_0_ on what occurred in universities for the 2020-2021 school year in which, to our knowledge, most, if not all, universities required facemasks in indoor public spaces.

Depending on the vaccine administered, preliminary clinical trials suggested efficacy ranging from 65–95% [10–12] against that alpha variant (B.1.1.7). As these statistics are derived by comparing the symptomatic case incidence in the vaccination group to that in the placebo group, it is likely that asymptomatic cases are missed in these statistics. Preliminary data suggests that vaccination reduces asymptomatic cases as well [28–31] and more recently [32]. The data in [30] was obtained from biweekly testing in healthcare workers and the author’s found the BNT162b2 vaccine 86% effective at preventing symptomatic and asymptomatic spread. Another study [31] analyzes biweekly screening tests in a group of employees at St Jude’s Childrens Hospital. A 72% reduction in asymptomatic cases was observed. The authors point out that short followup time, small cohort size, and that individuals choosing to not vaccinate might be higher risk could limit the accuracy of their findings.

During Summer 2020, just prior to reopening, the delta variant (B.1.617.2) became the dominant strain in the United States and broader global community. Preliminary studies suggest that vaccines, while effective at preventing hospitalization, are less effective at preventing infection from the delta variant [26, 32–35]. Conservative estimates put the effectiveness at preventing infection near 40%, while some studies have found effectiveness near 70%. An additional “booster” dose of the vaccine has been proposed to increase vaccine effectiveness. Preliminary studies from Israel suggest that such a strategy has a positive effect [36]. To model the current situation and the possibility of booster shots (perhaps in the Spring 2022 semester) we consider two different levels of vaccine effectiveness (50% and 80%) in our simulated scenarios.

In our model, vaccination and antibody status impact agents’ susceptibility and infectiousness. Each agent is assigned the *vaccinated attribute* independently with probability *V*. Such agents have *inward protection* factor *r*_*i*_ and *outward protection* factor *r*_*o*_. Vaccinated agents contribute a factor of 1−*r*_*o*_ of exposure time to susceptible agents in each meeting they are present at. When computing the probability vaccinated agents are infected at the end of a day, the probability is multiplied by 1−*r*_*i*_. All COVID-19 infections in vaccinated agents are classified as asymptomatic. We consider two levels of vaccine effectiveness: *r*_*i*_ = .5 = *r*_*o*_ and *r*_*i*_ = .80 = *r*_*o*_. The model is initiated with 10 randomly selected students in the exposed state. The main statistic we consider is the total percentage of agents ever in the exposed state i.e. the total number of infections divided by the population size 16800. We refer to this as the *percent infected*.

## 3 Results

**Fig 1:**
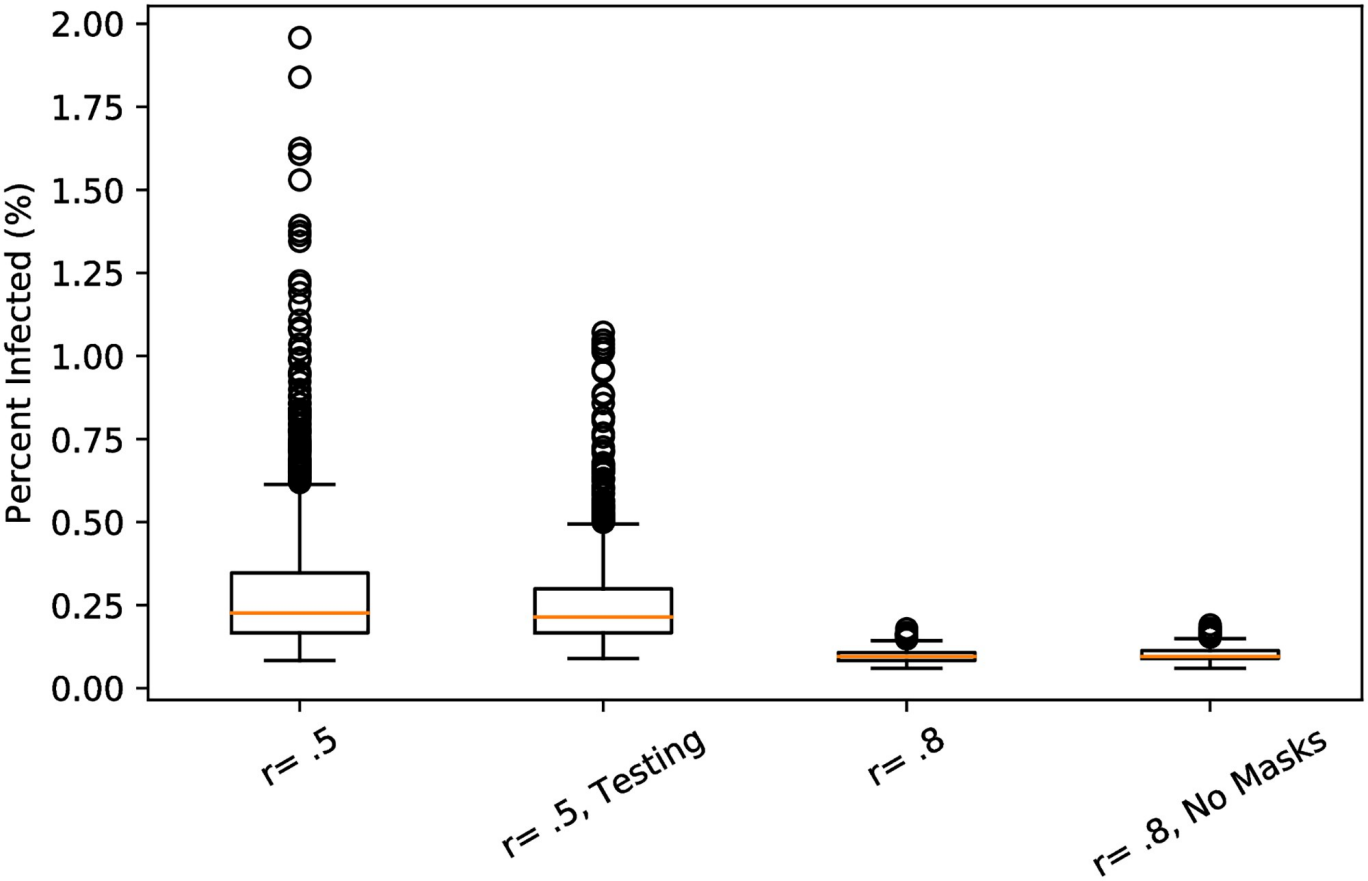
The percent of the population infected (*y*-axis) in 1000 simulated 15-week semesters per scenario. Each scenario assumes that 100% of the campus population is vaccinated. From left to right we consider the scenarios of: 50% vaccine effectiveness (*r* = .5), 50% vaccine effectiveness with screening testing (*r* = .5, Testing), 80% vaccine effectiveness (*r* = .8), and 80% vaccine effectiveness with no facemask use on campus (*r* = .8, No Masks).

Fig 1 displays box-plots with medians for the total percent of the campus population infected in 1000 simulated semesters in four different scenarios. In line with most universities policies, all campus participants are required to be vaccinated. Accordingly, we set vaccine coverage to *V* = 1.0 in all scenarios depicted in Fig 1.

The first scenario ‘*r*_*i*_ = .5’ is with 50% vacccine effectiveness so that *r*_*i*_ = 0.5 = *r*_*o*_ and no other measures in place (besides the implicit facemask use). The median total percent is .24. However, there is a strong right skew present in the data. We observed many simulations with more than 1% (168 total infections) and some simulations with nearly 2% (over 300 total infections) of the campus population infected.

The second scenario ‘*r* = 0.5, Testing’ again sets *r*_*i*_ = 0.5 = *r*_*o*_, but includes weekly screening testing of 25% of the campus population. While this did not lower the median percent infected compared to the scenario without testing, the right skew is less extreme. The bulk of outliers had less than 1% of the total population becoming infected.

The third scenario ‘*r* = .8’ increases vaccine effectiveness to 80% compared to the first scenario so that *r*_*i*_ = .80 = *r*_*o*_. We see that more effective vaccines significantly controls total infections with the median percent infected approximately 0.1% (18 infections). The right skew is also muted. The maximum number of total infections observed as 35 (0.2%).

The fourth scenario depicts the case of 80% vaccine effectiveness, but with no facemask use. Recall that our usual model assumes that facemasks are worn at all times in non-social settings. It has been estimated that facemasks, when worn by both infected and susceptible agents, reduce the susceptible agents exposure to the virus by at least 50% [37, 38]. To model no facemask use, we increase the duration of time spent in classrooms, the broad environment, clubs, and residence halls by a factor of 3. Tripling the duration triples the probability agents become infected in each interaction in these spaces. We observe that these increases do not cause for many more total infections. The median total infections remains bounded by 20 total infections (0.1%) and right tail events do not amount to many additional infections. In fact, over 1000 simulations we observed similar maximum total infections (34 versus 35) in the model with no multiplying factor and that with the risk multiplied by 3.

**Fig 2:**
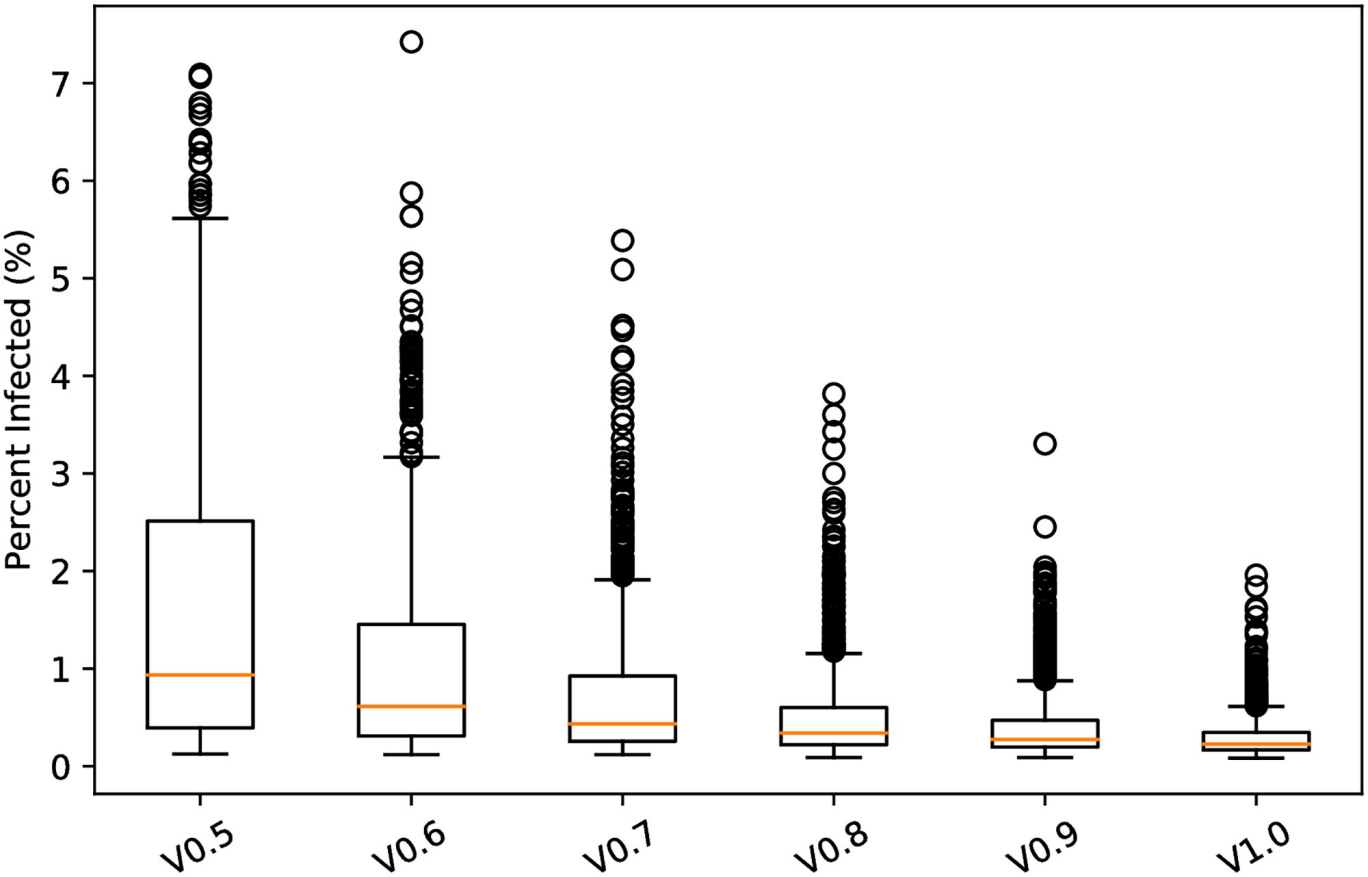
The percent of the population infected (*y*-axis) in 1000 simulated 15-week semesters per scenario. Each scenario assumes that 50% vaccine effectiveness and considers different proportions *V* ∈ {.5, .6, …, 1} of the campus population vaccinated.

Next, Fig 2 displays the percent infected in our model campus with varying vaccine coverage *V* ∈ {0.5, …, 1.0}. While increasing vaccine coverage reduces total infections, a striking feature of the data is that all scenarios exhibit right-skew. We see multiple outliers; some simulations in have over 7% of the campus population infected (1200 total infections).

## 4 Discussion

Given the emerging evidence that mRNA vaccines are less effective against preventing infection against the COVID-19 delta variant, our results suggests that caution is needed. Even with high levels of vaccine coverage, vaccinated members of the campus population may still become infected. Moreover, the right skew we observe for the percent infected implies that the risk of many agents in the model becoming infected is non-negligible.

Most infections in our model are asymptomatic cases. The reason for this is twofold. First, asymptomatic cases are more common in young people [39, 40]. Secondly, COVID-19 vaccines have been demonstrated to reduce symptomatic cases more effectively [10–12, 41]. CUNY campuses have a large proportion of minority groups. The 2019 CUNY Student Data Book reports a 25% Black and 30% Hispanic undergraduate population across all 25 CUNY campuses. Minority groups exhibit more vaccine hesitancy [19] and neighborhoods with more minority residents have higher COVID-19 incidence [42]. As many students at a non-residential urban university live at home, high levels of asymptomatic cases pose a silent risk to their households and communities.

Ensuring that a large proportion of the campus population is vaccinated, and administering screening testing are effective ways to control total infections. Most of the infections in our model are from socializing. Accordingly, students should be encouraged to practice safe social contact during the semester such as distancing and wearing facemasks in the presence of unvaccinated students. This is similar to what was suggested in [8]. We further comment that infections will lower at least proportional to the level of dedensification employed by the university. For example, if half as many students are regularly on campus we expect that total infections will reduce by at least half.

On a positive note, our study suggests that, so in the scenario that if the entire campus populations receives booster shots that raise vaccine efficacy above 80% for preventing both symptomatic and asymptomatic infections, then a safe university reopening with minimal extra precautions needed. Our model typically assumes that individuals use facemasks while participating in campus activities. However, additional simulations suggest that facemasks are not necessary with 80% vaccine effectiveness and 100% vaccine coverage.

A limitation with our model is the difficulty with setting parameters. Another is in designing the contact structure and relative risk of different meeting types. Socializing plays a dominant role for infection spread in our model, but it is difficult to create a realistic contact structure. A novel aspect of our approach compared to other agent-based COVID-19 university models [7, 8] is that we use a Markov chain to create social groups matching students with similar characteristics (year and area of study). Our sensitivity analysis also includes scenarios with less socializing. On a different note, if screening testing is present on campus, then there is the opportunity for college administrators to respond in real-time to rising case counts. For example, moving to all remote instruction when a certain threshold is reached. For that reason, testing may be even more effective than our simulations suggest.

Our model has many simplifications that could be refined. We assume constant vaccine effectiveness. Recent evidence suggests that vaccine effectiveness decreases over time [43]. Thus, future modeling could include a time-component for vaccine effectiveness. It would also be more accurate to start with heterogeneous vaccine effectiveness in the initial population (representing different vaccination times for agents and also additional (booster) vaccinations.) Another simplification is that we assume vaccinated and unvaccinated agents in the exposed state are equally likely to develop symptoms or be asymptomatic. Current evidence strongly supports less severe symptoms for vaccinated agents [44]. Lastly, our model does not account for different behavior among vaccinated individuals. It is plausible that vaccinated students socialize more and that students tend to socialize with others of similar vaccination status [45]. This would be interesting to incorporate and study further.

We conclude our discussion with a brief retrospective look (as of May 2022) at some COVID-19 Pandemic developments relevant to our modeling efforts. Most academic institutions (including CUNY) required, with a few exceptions, all students to vaccinate [46]. Speaking to CUNY specifically, facemasks were required on campus and random screening testing was in effect. Baruch College and other CUNY campuses changed many classes to fully remote or hybrid models. Consequently, the campus population was significantly dedensified. Between August 25th, 2021 and December 31st, 2021 CUNY administered approximately 95000 tests in the campus population and observed a positivity rate of 0.5% (475 cases) [47]. This outcome is consistent with our predictions in Fig 1 that assume 50% vaccine effectiveness. A major development late in the Fall 2021 semester (which primarily impacted the Spring 2022 semester) was the appearance of the omicron variant against which vaccines were significantly less effective, but symptoms were generally mild for vaccinated individuals [48]. Broadly, the attitude of living with the COVID-19 virus has become more and more prevalent. This has been accompanied by policy and behavioral changes [49]. We note that by updating contact structure, infection parameters, and agent behavior, our model is sufficiently flexible to capture aspects of such developments.

## 5 Conclusion

We constructed an agent-based SEIR model made to resemble a mostly non-residential urban university campus. We then ran different scenarios for vaccine effectiveness and coverage by the campus population. Given 50% vaccine effectiveness, it appears that additional interventions are needed for a safe reopening even with complete vaccine coverage. A right-skew for total infections suggests that rare but extreme events could have particularly bad outcomes. We found that screening testing helps control the variance in total infections. If the vaccines are 80% effective at preventing both symptomatic and asymptomatic COVID-19 infections, then our study suggests that no extra precautions are needed for a safe reopening.

## 6 Appendix

### 6.1 Model specifics

We utilize the agent-based campus Susceptible-Exposed-Infected-Removed model from [21]. Similar to [7, 8], students and faculty are assigned individualized schedules that they follow throughout a simulated semester. Schedules are organized into common meetings—classroom, broad environment, clubs, residential, socializing—during which COVID-19 is equally likely to be passed from infected agents present to each susceptible agent also present.

We use a standard agent-based SEIR model that considers each student as an agent who interacts through meetings. Meetings represent groups of students co-inhabiting and interacting in an activity such as class time, extracurricular, and socializing. All individuals in a meeting have equal exposure to one another for the entire meeting duration. Agents are either in susceptible, exposed, infectious, or removed states. We further stratify these states by vaccination status. In the next paragraph we describe how agents progress through these states. Broadly, an initial state is given to each agent and we measure how agent states evolve during one semester. The main difference of this approach from traditional agent-based SEIR models is that our contact structure is induced by the durations and intensities of the interactions through meetings.

All agents in the model start in either the susceptible state or with antibody protection. At the onset of the model, we independently assign each agent the *antibody attribute* with probability 0.20. A proportion of the agents with this attribute have *antibody protection* which prevents infection. Those without antibody protection act as normal susceptible agents. If a susceptible agent becomes exposed to COVID-19 then, after a random *incubation period*, the agent progresses to the asymptomatic or symptomatic infected state with equal probability [39, 40]. Such agents occupy this state for a random *infectious period* and subsequently transition to the recovered state. Symptomatic individuals decide after a random *observation period* to self-quarantine, either voluntarily or from seeking independent testing, until recovered. Recovered agents cannot become infected again. Except for the quarantine period, periods are modelled with independent geometric random variables. We write Geometric(1/*p*) to denote the geometric distribution *P*(*X* = *k*) = (1 − *p*)^*k*−1^ *p* for integers *k* ≥ 1 and 0 < *p* < 1 which has mean 1/*p*.

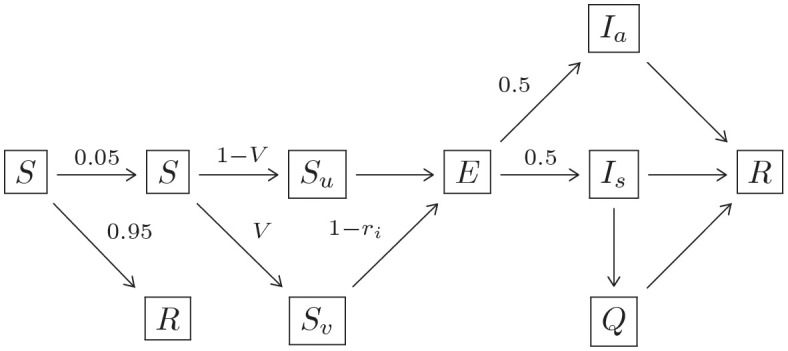
(1)

COVID-19 infections spread during meetings. Meetings include classes; the broad campus environment such as: hallways, elevators, lobbies, dining halls; time in dorms; clubs; and socializing. Each meeting occurs on a time and date and has a set *duration* in minutes. Duration is a measure of risk-intensity rather than time elapsed during a meeting. We scale it by the number of agents in the meeting and the risk of infection spread in the meeting type. For example, socializing has a longer duration than class time, not because more time is spent doing so, but because it is a riskier setting for infection spread [4, 50]. Complete details regarding meeting structure are in Section 6.2.

Each individual in a meeting in the susceptible state acquires exposure time equal to the meeting duration times the number of attendees in the infected state also at the meeting. At the end of each day, the total number of exposure minutes for each susceptible agent is tallied. This total is scaled by the *infection rate* which results in the probability the agent becomes infected on that day. The other manner in which infections occur in our model is through exogenous exposure in the non-campus community. We set the *average exogenous exposures per week* by applying a fixed (small) probability of becoming infected to each agent at the end of each day. Our model has 2 exogenous infections per week on average. Given our model’s population of 16800 agents, this corresponds to 1.7 positive cases per 100,000 agents per day. At the time of writing, New York City has a rate roughly 10 times this, but we expect the rate to drop by Fall 2021 [42].

The infection rate in our model is set to obtain an *average reproduction number* *R*_0_ = 3, which represents the average number of infections caused by an exposed agent in an entirely susceptible population. Estimates for COVID-19 spread in large communities (such as cities and countries) put *R*_0_ in the range [2.0, 3.0] [22, 23–25, 51], but there are some higher estimates [52]. The statistic varies by community contact structure. It has been observed that *R*_0_ is larger in reopened universities [4]. For example, [7] sets *R*_0_ = 3.8 in their campus COVID-19 model for a mostly residential urban university. See [7, 8] for more discussion about elevated *R*_0_ levels in a university setting.

**Fig 3:**
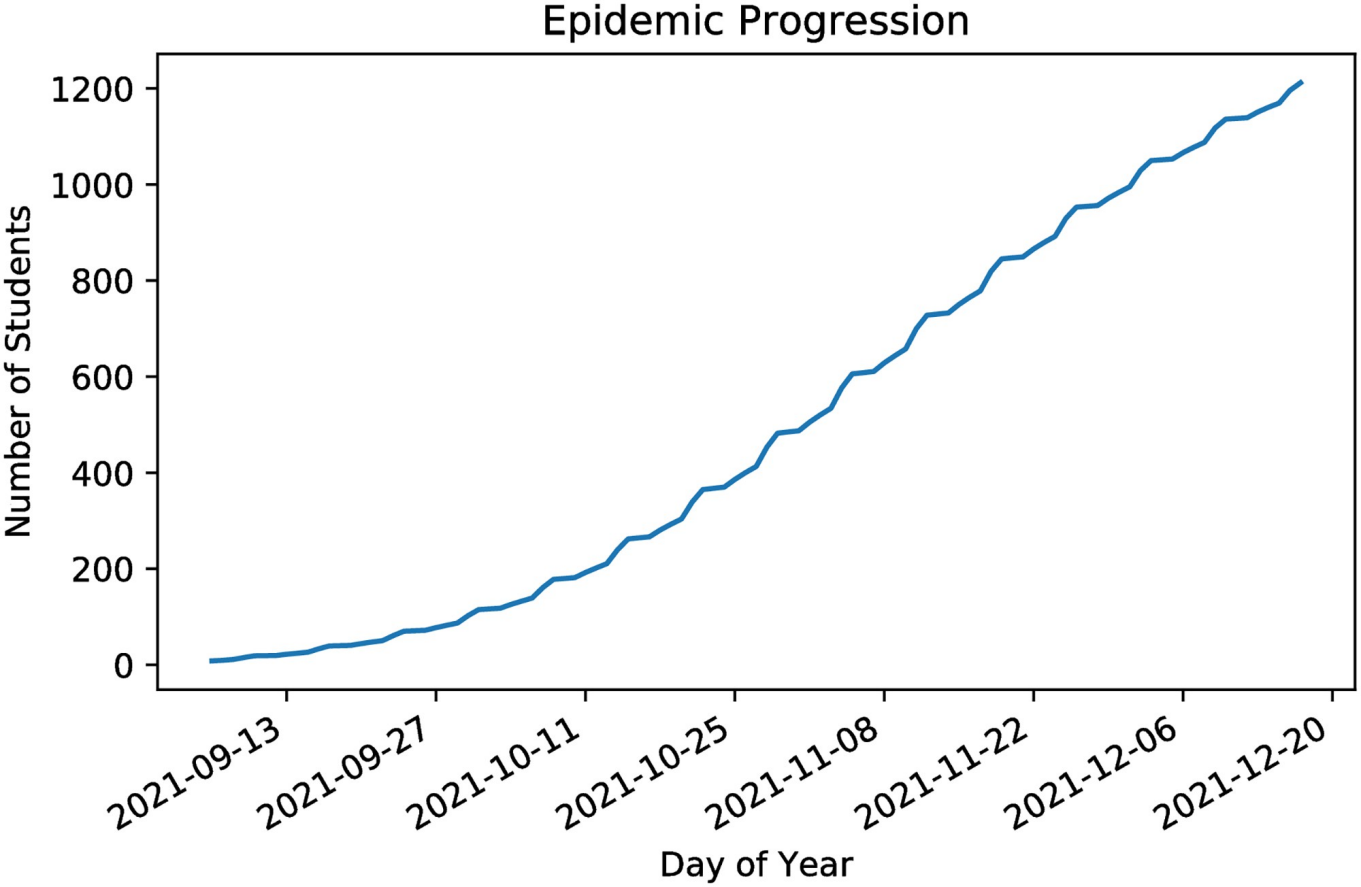
The number of infections over time in our base model with *R*_0_ = 3 and no vaccination.

**Fig 4:**
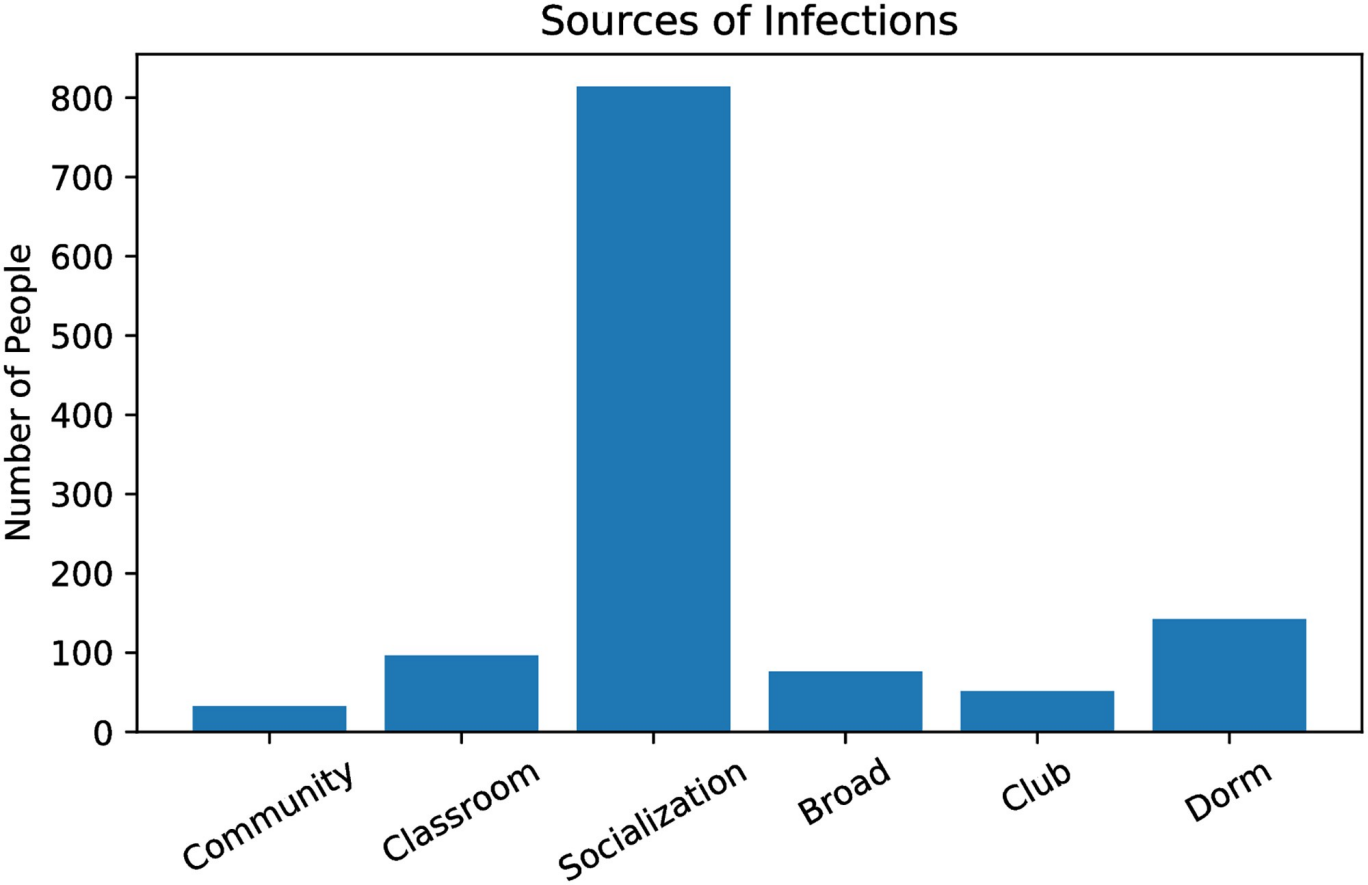
Total infections by source in our base model with *R*_0_ = 3 and no vaccination.

The main statistic we consider is the total number of agents ever in the exposed state over the course of a 15-week semester. We refer to this as *total infections*. The model is initiated with 10 randomly selected students in the exposed state. Our *base model* represents a reopening with the full population present on campus, antibodies present in 20% of the population, but no vaccination and no screening testing. We assume that facemasks are used except when socializing in private. This is accounted for by lowering the risk of infection spread in public spaces such as classrooms and broad environment. In the base model there is no active monitoring of the number of cases, so no adaptive policies (such as temporary suspension of in-person instruction) are ever implemented. With *R*_0_ = 3, we find, on average, 1200 total infections in the base model with no vaccination and 20% antibody incidence. Fig 3 gives a sense of how the number of infections evolves over time. Note that we do not include a “Thanksgiving Effect” with a November rise in infections in our model. Fig 4 shows the average number of infections occurring in each setting. As mentioned previously, socializing is the main venue for infection spread in our model. Note that we have 400 students living in the residential dorms. This is in alignment with Baruch College and amounts to less that 2% of the student population.

Vaccination and antibody status impact agents’ susceptibility and infectiousness. Each agent is assigned the *vaccinated attribute* independently with probability *V*. Such agents have *inward protection* factor *r*_*i*_ and *outward protection* factor *r*_*o*_. Vaccinated agents contribute a factor of 1−*r*_*o*_ of exposure time to susceptible agents in each meeting they are present at. When computing the probability vaccinated agents are infected at the end of a day, the probability is multiplied by 1−*r*_*i*_. All COVID-19 infections in vaccinated agents are classified as asymptomatic. Our medium-effectiveness scenarios set *r*_*i*_ = 0.5 = *r*_*o*_, while low-effectiveness has *r*_*i*_ = 0.2 = *r*_*o*_ and high-effectiveness has *r*_*i*_ = 0.8 = *r*_*o*_. We perform a sensitivity analysis to setting *r*_*i*_ ≠ *r*_*o*_ in Section 6.3. Table 1 shows the relevant infection parameters. Once exposed, vaccinated agents progress through the stages of COVID-19 infection (exposed, infectious, recovered) as a normal susceptible agent would. See (1) for a schematic and (Table 1) for details regarding time in each state.

**Table 1:** Infection parameters.

Parameter	Value	Parameter	Value
Incubation period	Geometric(3) days	Exogenous	2 weekly
Infectious period	Geometric(5) days	Antibody attribute	0.20
Observation period	Geometric(2) days	Antibody protection	0.95
Quarantine period	14 days	Inward protection	{0.2, 0.5, 0.8}
*R*_0_	3	Outward protection	{0.2, 0.5, 0.8}

### 6.2 Meeting structure

Each of the 16000 student is assigned a *Year* in 1,2,3,4 (in equal proportions) and an *Area* in Business, STEM, and Humanities. The proportion in each area is 75%, 15%, and 10%, respectively. The 800 faculty are divided in the same proportions as students to each of the three areas. The student/faculty designation, year and area of an agent play a role in the meetings they attends. Broadly, there are five types of meetings: class, broad, club, social, and residential. Students and faculty interact in class time and broad meetings. Only students interact in club, social, and residential meetings.

#### 6.2.1 Courses

Courses meet twice per week either MW or TuTh for *c* ⋅ 100/*L* minutes each class where *c* = 1/10 is a scaling parameter to account for reduced transmission probability in classrooms and *L* is the number of students enrolled. Courses are either General Interest (G), Business (B), STEM (S), or Humanities (H). Each class is independently designated as either a MW or TTh meeting class with probability 1/2 each. The number of classes of various sizes in Table 2 are chosen so that 20% of all classes are General Interest, and the proportions of classes of each size align with the counts provided by the Baruch College Common Data Set.

**Table 2:** Counts for various class sizes.

Class Size	10	20	30	40	50	75	150	(*C*_*X*_, *T*_*X*_)
G	8	40	140	60	30	40	10	(328, 13480)
B	24	120	420	180	90	120	30	(984, 40440)
S	5	24	84	36	18	24	6	(196, 8088)
H	3	16	56	24	12	16	4	(131, 5392)
**Total**	40	200	700	300	150	200	50	(1640, 67400)

We draw inspiration for how class meetings are generated in [7] using enrollment histogram data and order statistics to create correlations among courses in students among different years. Let *C*_*X*, *y*_ be the total number of classes of size *y* in area *X* ∈ {*B*, *S*, *H*, *G*}. For example, *C*_*B*,30_ = 420. Let *C*_*X*_ = ∑_*y*_ *C*_*X*, *y*_. Let $\vec{X}=\left(X_{1},\ldots ,X_{C_{X}}\right)$ be the sizes of classes in area *X* arranged from largest to smallest. For example

$$\begin{array}{c} \vec{S}=\left(\underset{C_{S,150}}{\underset{︸}{150,150,\cdots ,150}},\underset{C_{S,75}}{\underset{︸}{75,75,\cdots ,75}},50,\cdots ,10\right). \end{array}$$

Let $T_{X}=\sum_{i=1}^{C_{X}}X_{i}$ be the total number of seats offered across all of the courses in area *X*. Form the vector

$$\begin{array}{c} {\vec{p}}_{X}=\left(p_{1}\left(X\right),\cdots ,p_{C_{X}}\left(X\right)\right)\;\text{with}\;p_{i}\left(X\right)=\frac{X_{i}}{T_{x}}. \end{array}$$

Index the courses in $\vec{G}⊕\vec{X}≔\left(G_{1},\ldots ,G_{C_{G}},X_{1},\ldots ,X_{C_{X}}\right)$ as $\Omega_{X}=\left\{1,2,\ldots ,C_{G}+C_{X}\right\}$. Define the random variable *Y*(*X*) that takes values in *Ω*_*X*_ where, with probability 1/5, *Y*(*X*) is drawn from a multinomial with distribution ${\vec{p}}_{G}$ on $1,\ldots ,C_{G}$ and, with probability 4/5, is drawn from a multinomial with distribution ${\vec{p}}_{X}$ on $C_{G}+1,\ldots ,C_{X}$. We then assign classes to four students in area *X*, one of each year, simultaneously by sampling four independent *Y*_1_(*X*),…,*Y*_4_(*X*) ~ *Y*(*X*). Let

$$\begin{array}{c} Y_{\left(1\right)}\left(X\right)\leq Y_{\left(2\right)}\left(X\right)\leq Y_{\left(3\right)}\left(X\right)\leq Y_{\left(4\right)}\left(X\right) \end{array}$$

be the arrangement of the *Y*_*k*_(*X*) from least to greatest. The student in year *k* is assigned class *Y*_(*k*)_(*X*). Each student is assigned four classes in this manner.

This construction ensures that the amount of each class size in each area is proportional to the ratios in Table 2. Using order statistics ensures that students in an earlier year are more likely to take large, general interest classes. Faculty in the corresponding area are assigned to teach two uniformly samples courses in their area.

#### 6.2.2 Broad environment

All agents spend 20/*L* minutes per M, T, W, Th meeting with the *L* students and faculty in their area, and 10/16800 total minutes per week meeting with all agents in the model. This represents ambient environmental contacts (hallways, elevators, lobbies, gym, library) that occur on campus.

#### 6.2.3 Clubs

Clubs meet 100/*L* minutes on Thursday where *L* is the size of the club. There are 50 General Interest, 30 Business, 20 STEM, and 10 Humanities clubs. Each student joins a uniformly random general interest club with probability 1/5 and a uniformly random club in their area with probability 1/5. The probability a student does not participate in any clubs is (4/5) * (4/5) = 16/25 = 0.64. This is in line with the participation rates for clubs at Baruch College according to the 2018 Student Experience Survey.

#### 6.2.4 Residence hall

Pick 400 total students uniformly at random from years 1 and 2 to live in residence halls. Pair these students up into 200 groups of two students each representing roommates. Each roommate group meets 300 minutes per day. The entire group of 400 students in the residence hall spend 100/400 minutes together per week.

#### 6.2.5 Social

Small and large social groups are formed via a Markov process. All students are labeled as low, medium, or high socializers. In line with socializing surveys from [53], the probability a student is a low socializer is 0.15, medium is 0.45, and high is 0.40.

Let L,M, and H be the sets of low, medium, and high socializers. Furthermore, let *X*_*k*_(*Y*) be the set of level *Y* socializers from area *X* in year *k*. For example, $H_{3}\left(M\right)$ are medium-socializers in their third year of humanities. Whenever a student is sampled from a group *Z*, the sampling is done so that the student is uniformly sampled from Z∩L with probability 0.10, from Z∩M with probability 0.30, and from Z∩H with probability 0.60. Call this method (*).

A *small social group* is formed according to the following algorithm.

1. Select a student from the entire population according to (*). Suppose they are from area *X* and year *k*.
2. The next student is sampled according to (*) from:
  - The entire student population with probability 1/6.
  - All students in year *k* with probability 1/6.
  - All students in area *X* with probability 1/6.
  - All students in area *X* and year *k* with probability 1/2.
3. With probability 1/2, no more members are added to the group. With probability 1/2 the algorithm continues using the year and area of the newly added member to generate the next choice via (ii) and (iii).

A *medium social group* is formed by replacing the probability of adding an additional member to the group at step (iii) with 9/10. Every Friday there is a *large social group* consisting of five uniformly randomly selected medium social groups. The duration is 2000/*L* minutes *L* the total number of people in the meeting. The long duration of large social groups is capturing the “superspreader phenomenon” observed on campuses during the 2020-2021 school year [4].

Small social groups have expected size 3. These model close friends who study, eat, and pass time together. Medium social groups have expected size 11 and large social groups have expected size 55. These model larger social gatherings such as parties or events.

Each small group meets with probability 1/2 on each weekday M, Tu, W, Th for 1000/*L* minutes where *L* is the size of the group. This makes a minute of socializing ten times higher risk than a usual minute. Each medium group meets with probability 1/2 on Th and F for 1000/*L* minutes where *L* is the number of people in the meeting. Large social groups meet for 1000/*L* minutes on F (with probability 1). These random choices are made for the first week and repeated for all weeks thereafter. The parameter *s* scales for the higher risk of infection transmission during socializing since facemasks and social distancing are less likely to be employed. 100 is chosen so that the scaling is relative to the meeting time of a course. We form 3000 small social groups, 300 medium social groups, 50 large social groups for the base model.

### 6.3 Additional sensitivity analysis

**Fig 5:**
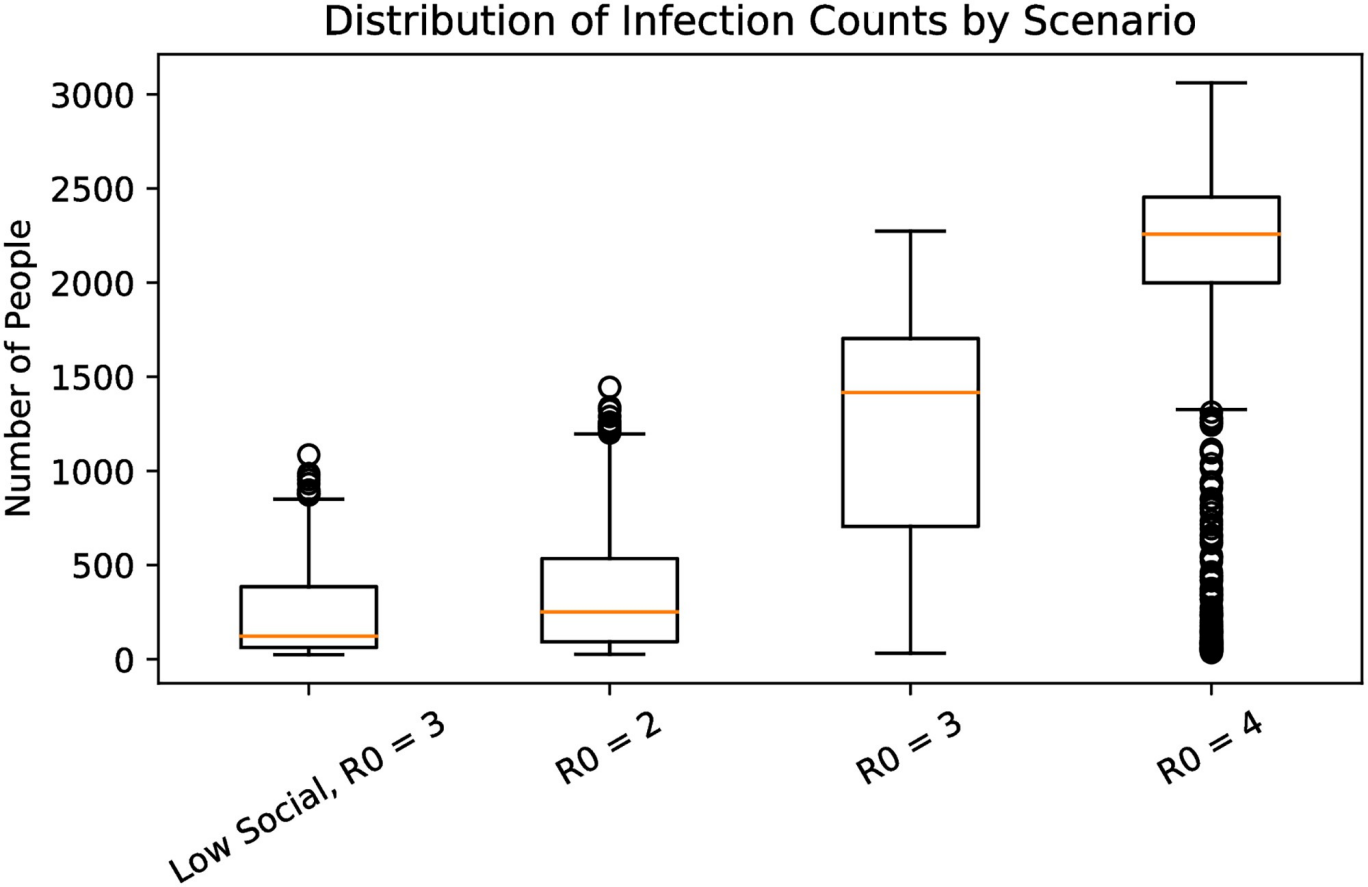
Total infections with *R*_0_ = 3 and socializing as in the base model as well as the base model with *R*_0_ = 2, 3, 4. The data is obtained from 1000 runs of each scenario.

**Fig 6:**
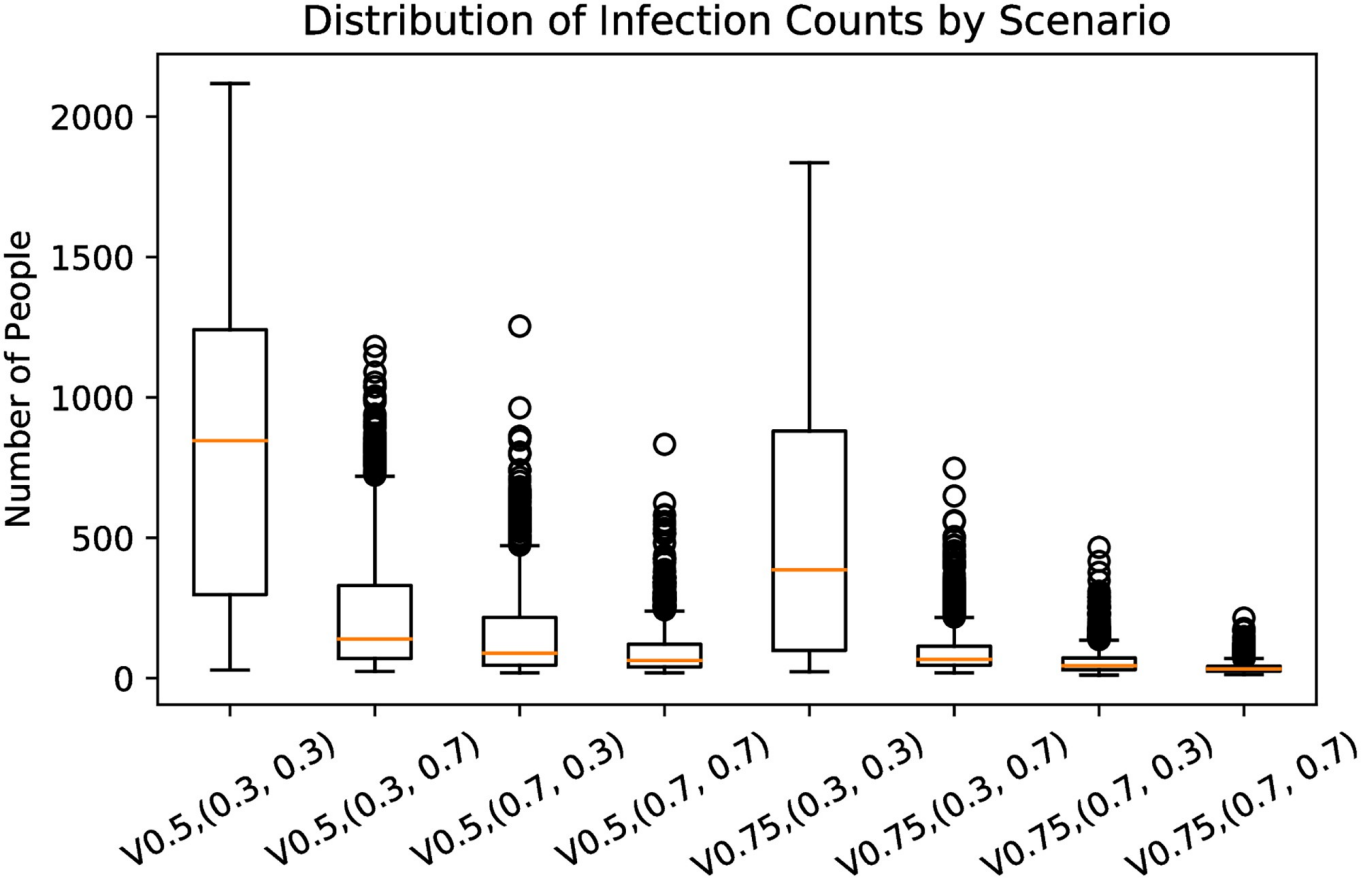
Total infections in the base model with different combinations of *r*_*i*_ and *r*_*o*_.

The reproduction number is a phenomenological output of the infection biology and contact structure in the model. Thus, it is is difficult to calibrate in heterogeneous populations (see the discussion in [8]). For this reason, we additionally run our base model with *R*_0_ = 2 and *R*_0_ = 4. Since socialization is a major source of infection spread, we also include a version with *R*_0_ = 3 and half as much social interactions. These variations are displayed in Fig 5. As expected, total infections are greatly reduced by decreasing socializing. Moreover, we see that total infections are sensitive to our choice of *R*_0_. This is more reason for administrators to exercise caution in their reopening plans. Lastly, Fig 6 shows box plots for total infections with unequal vaccine effectiveness parameters (*r*_*i*_, *r*_*o*_)∈{0.3, 0.7}^2^. We find that the impact from each is roughly the same. This suggests that our choices of setting *r*_*i*_ = 0.5 = *r*_*o*_ in our main analysis and also *r*_*i*_ = *r*_*o*_ in our low-, medium- and high-effectiveness vaccine scenarios reasonable simplifications to make.

### 6.4 Code access

The code for the project is publicly available on Github at the address https://github.com/MAS-Research/SEIR-Campus as an extension of [21]. There are two files associated with this paper: *CunyCovid.ipynb* replicates the simulations discussed in this paper and writes the data to a file. *ImageRendering.ipynb* loads the simulation data to produce the graphics shown in this paper.
